# Microfluidic Printing of Slippery Textiles for Medical Drainage around Wounds

**DOI:** 10.1002/advs.202000789

**Published:** 2020-06-11

**Authors:** Han Zhang, Guopu Chen, Yunru Yu, Jiahui Guo, Qian Tan, Yuanjin Zhao

**Affiliations:** ^1^ Department of Burns and Plastic Surgery Nanjing Drum Tower Hospital The Affiliated Hospital of Nanjing University Medical School Nanjing 210008 P. R. China; ^2^ Department of Clinical Laboratory Nanjing Drum Tower Hospital Clinical College of Xuzhou Medical University Nanjing 210008 P. R. China; ^3^ State Key Laboratory of Bioelectronics School of Biological Science and Medical Engineering Southeast University Nanjing 210096 P. R. China

**Keywords:** 3D printing, microfibers, microfluidics, wettability, wound healing

## Abstract

Surface materials with specific wettability play significant roles in existing fields from environmental protection to biomedicine. Here, a 3D droplet transport microfiber textile with slippery liquid‐infused porous surface is presented for medical drainage around wounds. The textile is fabricated by using a simple capillary microfluidic printing method to continuously spin polyurethane microfibers with liquid paraffin‐infused porous surface and print them into a 3D‐structure. Benefiting from the specific surface porous structure and oil encapsulation of the microfibers, aqueous droplets could be nondestructively and rapidly transported not only in simple single, double or multiple microfiber systems, but also in the microfibers composed stereoscopic textile through the microfluidic 3D printing. Based on this feature, it is demonstrated that the 3D slippery microfiber textile coupled with a vacuum sealing drainage therapy could significantly enhance the wound exudation drainage efficiency, reduce tissue injury, and prolong the effective service life in versatile wounds management. Thus, it is believed that the slippery microfiber textiles have potential for clinical applications.

## Graphical abstract

A 3D slippery microfiber textile from a microfluidic printing method can nondestructively and rapidly transport aqueous droplets in different dimensions. The textile with a vacuum sealing drainage therapy can thus enhance exudation drainage efficiency, reduce tissue injury, and prolong service life in versatile wounds management, which make it ideal for droplet manipulation in biomedical areas.

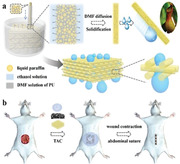

Surfaces with special wettability have recently aroused heated discussions due to their broad applications among fields ranging from environment,^[^ ^1^ ^]^ energy^[^ ^2^ ^]^ to biomedicine.^[^ ^3^, ^4^, ^5^, ^6^, ^7^ ^]^ Motivated by natural organisms such as lotus leaves,^[^ ^8^ ^]^ the legs of water striders,^[^ ^9^ ^]^ the Nepenthes pitcher plant,^[^ ^10^ ^]^ etc., a series of researches have focused on developing various functional surfaces with such special wettability.^[^ ^11^, ^12^, ^13^ ^]^ Among them, slippery liquid‐infused porous surfaces (SLIPSs) have received extensive attention for their stable and defect‐free repellency against multiple kinds of liquids or even liquid arrays.^[^ ^14^, ^15^, ^16^, ^17^ ^]^ With such an advantage, SLIPSs have shown great potential in droplet manipulation based applications.^[^ ^18^ ^]^ Although they have achieved great success in different areas like catheter drainage and biochemical analysis, etc.,^[^ ^19^, ^20^, ^21^ ^]^ the existing researches on SLIPSs are generally based on the planar films or the surface of massive solids, and thus greatly limiting their values in the fields that need complicated droplet manipulations. Therefore, the creation of a new slippery material for droplet manipulation in intricate dimensions is sorely demanded.

Here, we propose a novel slippery textile with the desired feature for medical drainage around wounds by using a microfluidic printing approach, as schemed in **Figure** **1**. As a promising and versatile method for synthesizing functional materials, microfluidic technology is widely used to prepare microparticles as well as microfibers.^[^ ^22^, ^23^, ^24^, ^25^, ^26^ ^]^ The products are with highly homogeneous morphology and controlled characteristics because the microfluidics can precisely deal with small volumes of fluids in integrated flow channels.^[^ ^27^, ^28^, ^29^, ^30^, ^31^ ^]^ For further creation of functional materials and performing complex applications, the versatility allows microfluidics to combine other strategies like flow lithography, digital controlling technique, 3D printing, etc.^[^ ^32^, ^33^, ^34^ ^]^ The resultant creations are endowed with abilities of highly precise and adjustable shapes, thus they could be applied in liquid biopsies, organs‐on‐chip, medical diagnostics, environment analyses, etc.^[^ ^35^, ^36^, ^37^ ^]^ However, to our knowledge, both of the microfluidics and printing technologies have not been utilized in the fabrication of slippery materials. Therefore, slippery materials with specific morphologies and functionalities are highly anticipated by using such novel technologies.

**Figure 1:**
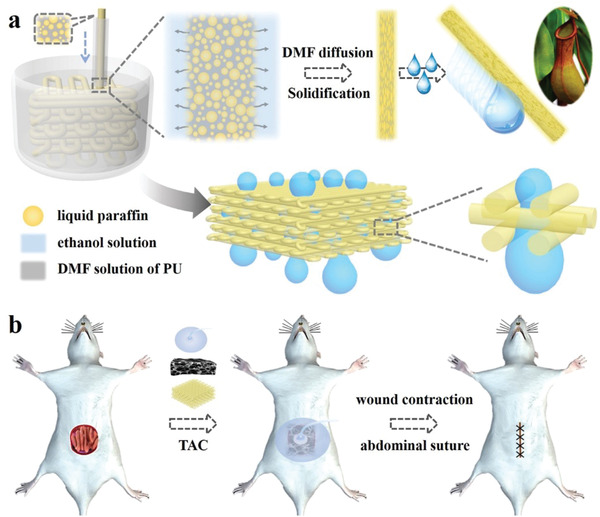
a) Scheme of fabrication process and wettability of the 3D‐structured slippery PU textile. b) Scheme of the 3D‐structured slippery textile coupled with a VSD device used in temporary abdominal closure (TAC) for wound repair.

In this paper, by employing a simple capillary microfluidic printing method to spin slippery polyurethane (PU) microfibers and print them into 3D‐structured textiles, we achieved the desired slippery textile for medical drainage. The slippery PU microfibers were formed by dispersing liquid paraffin emulsions into the PU pre‐spinning solution for imparting the resultant microfibers with oil‐infused porous surface morphology. Benefiting from the high specific surface areas of the porous structure and slippery property of the filled liquid paraffin, nondestructive and rapid transport of aqueous droplets were observed in single, double or multiple microfiber systems. It was also found that when these SLIPS microfibers were printed into 3D‐structured textile, it could still exhibit excellent slippery capability, enabling the multidirectional droplet transport in complex dimensions. Thus, the 3D slippery microfiber textile coupled with a vacuum sealing drainage (VSD) therapy could significantly enhance the wound exudation drainage efficiency, reduce tissue injury, and prolong the effective service life in versatile wounds management. These features make the slippery microfiber textile ideal for droplet manipulation in biomedical areas.

In a typical experiment, a capillary microfluidic system was used for continuous generation of slippery PU microfibers. The PU solution and the liquid paraffin were mixed in *N*, *N*‐dimethyl formamide (DMF) before they were pumped into the tapered cylindrical capillary microfluidic system. The stream was then flowed into a collection pool full of ethanol solution for fast diffusion of DMF to induce solidification of the PU microfibers (**Figure** **2**a‐i). During this period, PU microfibers with liquid paraffin emulsions could be continuously obtained (Figure 2a‐ii). After collection from the vessel, the microfibers showed a cylindrical structure and high uniformity, which was highly replicated the morphology of the stream (Figure 2a‐iii). Subsequently, the microstructures of the resultant PU microfibers were characterized by using a scanning electron microscope (SEM), as shown in Figure 2a‐iv–vii. It was demonstrated that the PU microfibers had not only porous surfaces but also interconnected pores after the liquid paraffin emulsions were removed. This manifested that the liquid paraffin was uniformly distributed on the surface of microfibers due to its low surface energy and water‐insoluble property in the process of DMF dispersion, and at the same time it has been filled inside the microfibers during the fabrication process.

**Figure 2:**
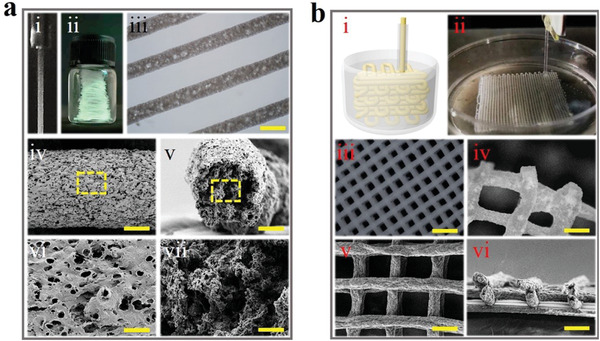
a) PU microfiber generated from microfluidics. i) Online spinning process of the microfiber. ii) Digital image of continuous microfibers in a vessel. iii) Optical microscopic image of the PU microfiber. iv–vii) SEM images of PU microfiber at iv) surface and v) cross section, and the magnified field of vi) surface as well as vii) cross section. Scale bars are iii) 200 µm, iv,v) 50 µm, vi,vii) 5 µm. b) 3D‐structured slippery PU textile. i) Schematic 3D printing fabrication process. ii) Digital image of the generating process. iii,iv) Optical microscopic image of iii) the overall morphology of PU textile and iv) its connection region. v,vi) SEM images of PU textile at v) surface and vi) cross section. Scale bars are iii) 1 mm and iv–vi) 200 µm.

Furthermore, by using a microfluidic 3D printing method, adjusting the flow rate of fluid to match the movement speed of the nozzle of the 3D printer, these soft and long PU microfibers could be weaved into a 3D‐structured textile (Figures S1 and 2b‐i–iii, Supporting Information). Because the diffusion rate of DMF was slower than the weaving rate of the microfiber, the solidification of the microfiber did not complete when it was constructed into the 3D structure, resulting in the good connections between each layer of the textile that it could not be easily separated (Figure 2b‐iv). From the SEM images of the textile, it could be found that the textile could not only keep the same structure as it was printed but also maintain the characteristics of the single slippery microfiber (Figure 2b‐v,vi). Benefiting from the controlled flow rate of pre‐mixed solution and orifice diameter of the capillary, the diameter of the resultant PU microfiber could be easily tailored (Figure S2, Supporting Information). Similarly, the programmable manipulations of microfluidic printing process enabled the feasible control over shapes and sizes of the textile (Figure S3, Supporting Information). It could be shown that various shaped textiles ranging from a nonangular heart to a polygonal shape with more angularities could be achieved. In order to explore the application value of the fabricated slippery PU microfibers and textiles, tensile tests were conducted. It was found that both the slippery PU microfibers and textiles had good tensile property due to their porous structure, as shown in Figures S4–S7 (Supporting Information).

Taking advantage of the high specific surface area coming from the porous structure of the microfiber and slippery property of the filled liquid paraffin, the resultant microfibers exhibited typical properties of the SLIPS, such as low surface free energy and low adhesion force, etc., which were important factors for nondestructive and rapid aqueous droplets transport (Figure S8, Supporting Information). This was firstly observed on a single microfiber, where liquid of virtually any surface tension was repelled on the surface of the microfiber and rapidly slipped down the microfiber (**Figure** **3**a and Movie S1, Supporting Information). The slip velocity of different kinds of liquids including deionized water and other liquids such as PBS buffer and simulated blood at different tilt angles was also measured, where an increased tilt angle of the microfiber resulted in an increased slip velocity. (Figure 3d and Figure S9, Supporting Information). Moreover, the transport ability of droplets in two microfibers systems was also investigated (Figure 3b and Movie S1, Supporting Information). It could be found that the droplet slid down along the upper microfiber smoothly without being disturbed by the node due to low adhesion, and this would not be affected by different assembly nodes and the angles (Figure S10a,b, Supporting Information). To demonstrate the droplet transport ability of these microfibers in 3D space environment, multiple slippery microfibers were interweaved (Figure 3c and Movie S1, Supporting Information). It was worth mentioning that the microfiber located on the uppermost layer had a certain angle with the plane formed by the first two crossed microfibers to construct stereoscopic space. The result was similar to that in single or two microfiber system, where the droplet slipped down along one microfiber and the slip process would not be interfered by the node. Also, the slip velocity in these different node‐angle microfibers systems were studied (Figure 3e and Figure S10c, Supporting Information), which showed the same trend as that in the single microfiber system. The above results showed that the droplets could slide down unimpededly in different microfiber‐assembled systems, indicating the 3D droplet manipulation ability of these assembled PU microfibers.

**Figure 3:**
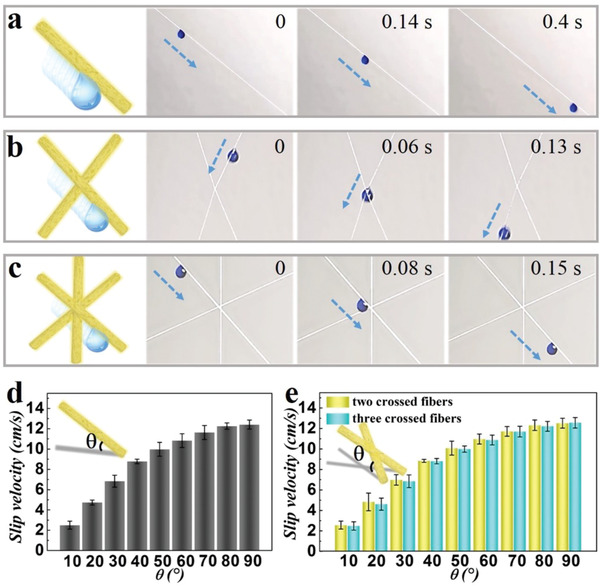
a–c) Scheme and real‐time images of aqueous droplet slipping in a) single, b) 30° crossed double and c) multiple microfibers system. d,e) The relationships between slip velocity of droplet and d) tilt angle of single microfiber, e) inclination angle of two crossed (yellow) as well as three crossed (blue) fibers.

Since the slippery textiles were generated by assembling the microfibers through 3D printing technology, each contact surface of the 3D stereoscopic structured textiles was endowed with superb slippery property. The surface wettability of the textile was first investigated. It was found that the aqueous droplet contact angle on the surface of slippery textiles with liquid paraffin‐infused was 86.355° while that of the common textiles without liquid paraffin infused was 114.008° (**Figure** **4**a). Besides, the sliding angle of the aqueous droplet was only 6° on the slippery textiles but increased to 85° on ordinary textile, indicating the slippery textile had a better droplet manipulation property. In addition, the water contact and sliding angle on the surface of slippery textile remained almost unchanged after textile being immersed in the PBS buffer for 8 days (Figure S11, Supporting Information). Notably, 3D droplet transport capabilities of the slippery textile were also investigated. First, no residue was found when the droplet slid down the surface of the inclined slippery textile (Figure 4b‐i and Movie S2, Supporting Information), which should be resulted from the fact that the slippery surface of each microfiber would verify the unimpeded transport of the aqueous droplet. Moreover, the slippery textile showed excellent performance in stereo transport of aqueous droplet (Figure 4b‐ii and Movie S2, Supporting Information). It could be found that a large aqueous droplet infiltrated through the textile smoothly without any residue under the existence of a negative pressure attraction. This should be ascribed that the slippery surfaces and good connection between microfibers allowed the successful and non‐destructive droplet transport in the 3D construct.

**Figure 4:**
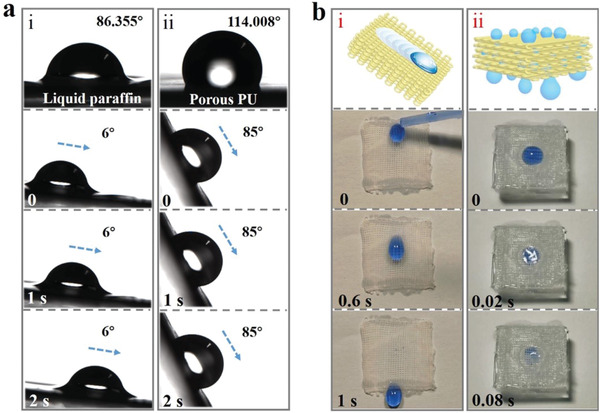
a) Contact angles and droplet sliding images of i) liquid paraffin infiltrated porous PU textile and ii) common porous PU textile without liquid paraffin infused. b) Schematic and real‐time images of the droplet i) slipping on the inclined slippery PU textile surface, and ii) infiltrated through the PU textile under the existence of a negative pressure attraction.

To demonstrate the practical value of the slippery textile, it was used for managing severe surface wound. In this situation, large quantities of tissue effusion are produced continuously, leading to frequent change of wound dressings to avoid infections. Thus, vacuum sealing drainage technique is developed to replace wound dressings to reduce the burden on medical personnel.^[^ ^38^, ^39^ ^]^ However, the therapeutic effects of the VSD are limited by the fact that sponge applied to the wound surface may be polluted by impurities in exudation, and may induce bleeding when being removed as the granulation tissues could directly regenerate into the sponge. These drawbacks could be remedied by the assistance of slippery textile we present. Before application, biocompatibility of the slippery textile was investigated (Figure S12, Supporting Information). The results showed that the 3D printed slippery textile showed great biocompatibility. In addition, an infected full‐thickness skin defect rat model was constructed to simulate the wound effusion condition. Then, the slippery textile was placed between the PU sponge and wounded tissue, followed by subsequent negative pressure device. After 3 days of common VSD, the wound surface treated by slippery textile‐assisted VSD was well cleaned and drained, and the surface of the slippery textile was free of liquid or other impurities (Figure S13a and Movie S3, Supporting Information). During this period, the slippery textile could keep clean while enhance therapeutic effects by separating the contaminative sponge from the wound bed to avoid superinfection. To test the wound healing performance, Hematoxylin and eosin (H&E) staining and Masson staining were conducted after 1 week. It was suggested that the back wound healed well and the negative pressure device promoted angiogenesis (Figure S13b–e, Supporting Information).

Apart from surface wound, the value of the slippery textile assisted‐VSD on exposed organ was further evaluated. The Food and Drug Administration (FDA) has forbidden the application of VSD on exposed organ as the direct contact of sponge to organ surface may cause many complications, such as bleeding, infections, abrasion, etc. As an alternative, in the management of exposed bowels after open abdomen, a vacuum‐assisted closure system (V.A.C.) was developed with an additional micro‐perforated plastic film to separate sponge and bowels to avoid their direct contact. However, many countries cannot obtain the V.A.C. and the effects of plastic film are limited to some cases. In this paper, an open‐abdomen model was established through wiping out part of the abdominal wall to expose bowel to the air. Afterward, the slippery textile was applied to cover the whole bowels followed by the VSD, as shown in **Figure** **5**a. It was demonstrated that the effusion drainage process in the abdominal cavity was efficient (Figure 5b and Movie S3, Supporting Information). Hematoxylin and eosin (H&E) staining and Masson staining were conducted to test the protective effects of slippery textile on bowels. No abrasion was observed in the slippery textile‐assisted VSD group as serosa or muscle almost kept integrated. In contrast, in the common VSD group, the serosa and muscle of the bowel were broken severely and may be potential for fistula (Figure 5c). SEM images and optical images showed the same results (Figure S14, Supporting Information). These results indicated the slippery textile could performed excellently in versatile wound management applications.

**Figure 5:**
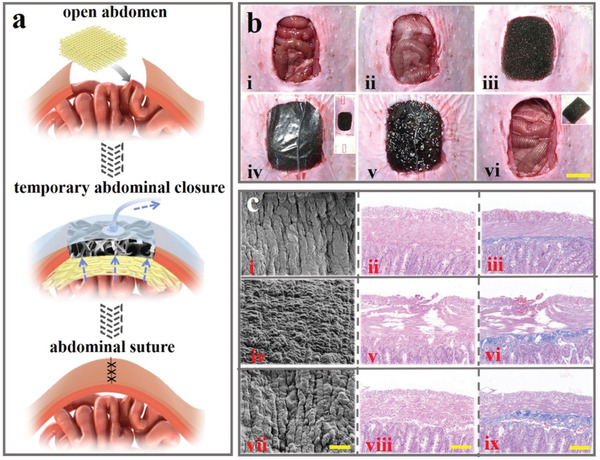
a) Scheme of slippery PU textile applied for abdominal wound repair. b) Images of repairing process using slippery textile‐assisted VSD. Scale bar is 1 cm. c) SEM, H&E staining and Masson staining images of i–iii) normal intestinal tissue, iv–vi) abraded intestinal tissue in common VSD condition, and vii–ix) protected intestinal tissue in slippery textile‐assisted VSD condition. Scale bars are i,iv,vii) 20 µm and ii,iii,v,vi,viii,ix) 100 µm.

In summary, we proposed a slippery textile for medical drainage by employing a simple capillary microfluidic printing method to spin slippery polyurethane microfibers and print them into 3D‐structured textiles. Due to the pre‐dispersion of liquid paraffin into PU solution, the resultant microfibers were imparted with oil‐infused porous surface morphology. Taking advantage of the porous structure and slippery property of the filled liquid paraffin, non‐destructive and rapid transport of droplets could be achieved on the surface of single, double, multiple microfibers. It was demonstrated that the multidirectional droplet transport in complex dimensions could be achieved when these SLIPS microfibers were constructed into stereoscopic structured textile through 3D printing technique. Consequently, the 3D slippery microfiber textile coupled with a VSD therapy could significantly enhance the medical drainage and treatment efficiency, reduce tissue injury, and prolong the effective service life in versatile wounds management. These features indicate the slippery microfiber textile will be the most promising candidate in droplet manipulation in biomedical fields.

In the recent decades, materials with specific surface properties inspired by nature creatures have been arousing wide concerns all over the world. Among them, SLIPSs have stood out in droplet transport due to their state‐of‐the‐art surfaces with stable and defect‐free repellency against multiple liquids or even liquid arrays. However, most researches were focusing on the planar films or the surface of massive solids and few experiments have been focusing on SLIPS with 3D structures. This limited the complex applications especially the biomedical applications in real life, which is much more complex than the existed droplet transport situations. Therefore, we proposed a 3D stereoscopic structured slippery textile from the capillary microfluidic printing method, which demonstrated their advantages over 3D droplet transport in medical drainage. In addition, more complex structures will be generated according to future demands. Based on these excellent characteristics, such slippery textile is also potential in pollution cleaning, biological detection, droplet collection, etc. To implement these values, future endeavors will be focused on improving their repeatability and realizing integration with other platforms.

## Conflict of Interest

The authors declare no conflict of interest.

## Supporting information

Supporting Information

Supplemental Movie 1

Supplemental Movie 2

Supplemental Movie 3

## References

[1] X. L. Tian , T. Verho , R. H. A. Ras , Science 2016, 352, 142. 27124437 10.1126/science.aaf2073

[2] N. Miljkovic , R. Enright , Y. Nam , K. Lopez , N. Dou , J. Sack , E. N. Wang , Nano Lett. 2013, 13, 179. 23190055 10.1021/nl303835d

[3] L. Aurélie , D. Quéré , Nat. Mater. 2003, 2, 457. 12819775 10.1038/nmat924

[4] X. Yao , Y. Song , L. Jiang , Adv. Mater. 2011, 23, 719. 21287632 10.1002/adma.201002689

[5] J. Wang , W. Gao , H. Zhang , M. Zou , Y. Chen , Y. Zhao , Sci. Adv. 2018, 4, eaat7392. 30225367 10.1126/sciadv.aat7392 PMC6140404

[6] R. Yudi , L. B. Xu , S. Yang , J. Mater. Chem. A 2013, 1, 2955.

[7] J. Wang , Y. Zhang , S. Wang , Y. Song , L. Jiang , Acc. Chem. Res. 2011, 44, 405. 21401081 10.1021/ar1001236

[8] Y. Zhao , C. Yu , H. Lan , M. Cao , L. Jiang , Adv. Funct. Mater. 2017, 27, 1701466.

[9] K. Yang , G. Liu , J. Yan , T. Wang , X. Zhang , J. Zhao , Bioinspiration Biomimetics 2016, 11, 066002. 27767015 10.1088/1748-3190/11/6/066002

[10] T. S. Wong , S. H. Kang , S. K. Y. Tang , E. J. Smythe , B. D. Hatton , A. Grinthal , J. Aizenberg , Nature 2011, 477, 443. 21938066 10.1038/nature10447

[11] Y. Wang , L. Shang , G. Chen , L. Sun , X. Zhang , Y. Zhao , Sci. Adv. 2020, 6, eaax8258. 32042897 10.1126/sciadv.aax8258 PMC6981080

[12] M. Zhang , P. Wang , H. Sun , Z. Wang , ACS Appl. Mater. Interfaces 2014, 6, 22108. 25418198 10.1021/am505490w

[13] X. Zhang , L. Sun , Y. Wang , F. Bian , Y. Wang , Y. Zhao , Proc. Natl. Acad. Sci. USA 2019, 116, 20863. 31570600 10.1073/pnas.1912467116 PMC6800372

[14] S. Amini , S. Kolle , L. Petrone1 , O. Ahanotu , S. Sunny , C. N. Sutanto , S. Hoon , L. Cohen , J. C. Weaver , J. Aizenberg , N. Vogel , A. Miserez , Science 2017, 357, 668. 28818939 10.1126/science.aai8977

[15] J. G. Kim , H. J. Choi , K. C. Park , R. E. Cohen , G. H. McKinley , G. Barbastathis , Small 2014, 10, 2487. 24648034 10.1002/smll.201303051

[16] J. Wang , L. Sun , M. Zou , W. Gao , C. Liu , L. Shang , Z. Gu , Y. Zhao , Sci. Adv. 2017, 3, 1700004. 10.1126/sciadv.1700004 PMC5457146 28630920

[17] J. Wang , Y. Huang , K. You , X. Yang , Y. Song , H. Zhu , F. Xia , L. Jiang , ACS Appl. Mater. Interfaces 2019, 11, 7591. 30673218 10.1021/acsami.8b21088

[18] K. Jun , H. Yabu , Adv. Funct. Mater. 2015, 25, 4195.

[19] P. Dibyangana , U. Manna , J. Mater. Chem. A 2018, 6, 22027.

[20] M. Cao , D. Guo , C. Yu , K. Li , M. Liu , L. Jiang , ACS Appl. Mater. Interfaces 2016, 8, 3615. 26447551 10.1021/acsami.5b07881

[21] C. Howell , A. Grinthal , S. Sunny , M. Aizenberg , J. Aizenberg , Adv. Mater. 2018, 30, 1802724. 10.1002/adma.201802724 30151909

[22] Y. Yu , F. Fu , L. Shang , Y. Cheng , Z. Gu , Y. Zhao , Adv. Mater. 2017, 29, 1605765. 10.1002/adma.201605765 28266759

[23] J. H. Kim , T. Y. Jeon , T. M. Choi , T. S. Shim , S. H. Kim , S. M. Yang , Langmuir 2014, 30, 1473. 24143936 10.1021/la403220p

[24] Q. Ma , Y. Song , J. W. Kim , H. S. Choi , H. C. Shum , ACS Macro Lett. 2016, 5, 666. 10.1021/acsmacrolett.6b00228 35614670

[25] G. P. Chen , Y. R. Yu , G. F. Wang , G. S. Gu , X. W. Wu , J. A. Ren , H. D Zhang , Y. J. Zhao , Research 2019, 2019, 6175398. 10.34133/2019/6175398 PMC6750103 31549071

[26] S. H. Kim , J. G. Park , T. M. Choi , V. N. Manoharan , D. A. Weitz , Nat. Commun. 2014, 5, 3068. 24394965 10.1038/ncomms4068

[27] C. Shao , Y. Liu , J. Chi , J. Wang , Z. Zhao , Y. Zhao , Research 2019, 2019, 9783793. 10.34133/2019/9783793 PMC6946249 31922149

[28] M. Qin , M. Sun , R. Bai , Y. Mao , X. Qian , D. Sikka , Y. Zhao , H. Qi , Z. Suo , X. He , Adv. Mater. 2018, 30, 1800468. 10.1002/adma.201800468 29638026

[29] Y. Song , T. C. T. Michaels , Q. M. Ma , Z. Liu , H. Yuan , S. C. Takayama , T. P. J. Knowles , H. C. Shum , Nat. Commun. 2018, 9, 1. 29844310 10.1038/s41467-018-04510-3 PMC5974351

[30] X. Zhang , Y. Yu , G. Chen , L. Sun , Y. Zhao , Sci. Bull. 2019, 64, 1110. 10.1016/j.scib.2019.06.016 36659772

[31] A. Shastri , L. M. McGregor , Y. Liu , V. Harris , H. Q. Nan , M. Mujica , Y. Vasquez , A. Bhattacharya , Y. T. Ma , M. Aizenberg , O. Kuksenok , A. C. Balazs , J. Aizenberg , X. M. He , Nat. Chem. 2015, 7, 447. 25901824 10.1038/nchem.2203

[32] D. B. Kolesky , R. L. Truby , A. S. Gladman , T. A. Busbee , K. A. Homan , J. A. Lewis , Adv. Mater. 2014, 26, 3124. 24550124 10.1002/adma.201305506

[33] A. K. Au , W. Huynh , L. F. Horowitz , A. Folch , Angew. Chem., Int. Ed. 2016, 55, 3862. 10.1002/anie.201504382 PMC7679199 26854878

[34] A. Polat , S. Hassan , I. Yildirim , L. E. Oliver , M. Mostafaei , S. Kumar , S. Maharjan , L. Bourguet , X. Cao , G. L. Ying , M. E. Hesaraj , Y. S. Zhang , Lab Chip 2019, 19, 550. 30657153 10.1039/c8lc01190g PMC6391727

[35] Y. S. Zhang , K. Yue , J. Aleman , K. M. Moghaddam , S. M. Bakht , J. Z. Yang , W. T. Jia , V. D. Erba , P. Assawes , S. R. Shin , M. R. Dokmeci , R. Oklu , A. Khademhosseini , Ann. Biomed. Eng. 2017, 45, 148. 27126775 10.1007/s10439-016-1612-8 PMC5085899

[36] J. T. Muth , D. M. Vogt , R. L. Truby , Y. Mengüç , D. B. Kolesky , R. J. Wood , J. A. Lewis , Adv. Mater. 2014, 26, 6307. 24934143 10.1002/adma.201400334

[37] Y. S. Zhang , A. Arneria , S. Bersini , S. R. Shin , K. Zhu , Z. G. Malekabadi , J. Aleman , C. Colosi , F. Busignani , V. D. Erba , C. Bishop , T. Shupe , D. Demarchi , M. Morett , M. Rasponi , M. R. Dokmeci , A. Atala , A. Khademhosseini , Biomaterials 2016, 110, 45. 27710832 10.1016/j.biomaterials.2016.09.003 PMC5198581

[38] H. Alakus , F. Popp , J. M. Leers , C. J. Bruns , A. H. Hölscher , W. Schröder , S. H. Chon , Surg. Endosc. 2018, 32, 1906. 29218673 10.1007/s00464-017-5883-4

[39] J. Wang , H. Zhang , S. Wang , Minerva Chir. 2015, 70, 17. 25389757

