# Supplementary material for: Microfluidic Printing of Slippery Textiles for Medical Drainage around Wounds
Source: Adv Sci (Weinh). 2020 Jun 11;7(16):2000789. doi: 10.1002/advs.202000789 (PMC7435260; doi:10.1002/advs.202000789)
Supplement: Supplementary file 1 — Supporting Information [file ADVS-7-2000789-s001.pdf]

Copyright WILEY-VCH Verlag GmbH & Co. KGaA, 69469 Weinheim, Germany, 2020.

Supporting Information

Microfluidic Printing of Slippery Textiles for Medical Drainage around Wounds

*Han Zhang, Guopu Chen, Yunru Yu, Jiahui Guo, Qian Tan, Yuanjin Zhao**

Experimental Section

Materials and animals: Polyurethane (PU) was purchased from Zhejiang Huafon Spandex Co., Ltd. N, N-Dimethylformamide (DMF), Dimethyl sulfoxide (DMSO), ethanol were obtained from Sinopharm Chemical Reagent Co., Ltd. (Shanghai, China). Liquid paraffin was purchased from Nanjing General Hospital of Nanjing Command, PLA. The 3T3 cells were obtained from the Cell Bank of Chinese Academy of Sciences (Shanghai, China). Calcein-AM was obtained from Molecular Probes Co. MTT reagent was purchased from J&K Scientific Ltd., Shanghai, and prepared 5 mg/mL in PBS. The concentration of ethanol solution in collection pool was 75% to reduce the density of the solution for preventing the PU slippery microfibers from floating. Phosphate Buffered Saline (PBS) solution was freshly prepared in our laboratory. Deionized water used in the whole experiments was obtained from a Millipore Milli-Q system (Millipore, Bedford, MA) with a resistivity of 18 MΩ·cm. Other chemical reagents were of the best grade available and used as received. The male Sprague-Dawley rats (8 to 12-week-old) were achieved from Nanjing General Hospital of Nanjing Command, PLA. (Nanjing, China). All rats were treated strictly according to the Laboratory Animal Care and Use Guidelines. All of the animal care and experimental procedures were reviewed and approved by the Animal Investigation Ethics Committee of Nanjing General Hospital of Nanjing Command.

Microfluidics: The capillary microfluidic device was composed of one cylindrical glass capillary tube and a piece of glass slide. The capillary with an original diameter of 800 μm was obtained from World Precision Instruments, Inc. The capillary was firstly tapered by a micropipette puller (Sutter Instrument Co., Novato, USA) and sanded under optical microscope to reach the desired orifice diameter of about 300 μm before it was immobilized on the glass slide with epoxy resin (Devcon 5 Minute Epoxy). To fabricate the microfibers, the PU solution was pumped into the device through syringe pumps (PHD 2000 series, Harvard, Plymouth Meeting, PA). The slippery PU microfibers with different sizes could be generated through different flow rates of the pre-mixed solution and different orifice diameters.

3D printing of textile: The 3D printer was purchased from Nanjing Additive Manufacturing Research Institute. The size and shape of the textiles were controlled by the 3D printed models, which were drawn by 3DMax software and stored in .stl format. In order to adapt to the speed of the syringe pump, the moving speed of the nozzle and the filling rate of the printed model were correspondingly changed. In addition, the thickness of the printed textiles could be controlled by adjusting the printing time. In this experiment, the syringe pump was set at a speed of 0.8 mL/h. The moving speed, filling rate and printing time of the 3D printer were set at 0.35 mm/s, 80% and 30 min, respectively.

Droplet manipulation performance test: The slippery microfibers of the same length were fixed, and then the droplets were added from a fixed height. The time they took for the droplets to slip down was recorded to measure the average slip velocity. By adjusting the angle between the microfiber and the table, the slip velocity under different tilt conditions was tested. In addition, multiple microfiber systems were tested in the same way. Among them, the assembly of the three microfibers was slightly different. The third microfiber was not in the same plane as the first two microfibers. Therefore, the inclination angle mentioned in the test was the angle between the third microfiber and the table.

Cell culture: The 3T3 cells were incubated within high sugar Dul-becco's modified Eagle's medium (High Glucose DMEM, Invitrogen, Nanjing and Biotech Development Co. Ltd) which were mixed with 10% (v/v) FBS (fetal bovine serum) and 1% (v/v) 10,000 U mL-1 penicillin-streptomycin double antibiotics in the incubator (HERA Cell 150, Thermo, USA) with 5% CO2 at 37 °C for growth and proliferation. All the fabricated textiles were immersed in 75% ethanol solution under UV light irradiation overnight to achieve sterilization. The normal and slippery PU textiles were then washed by sterile PBS solution for three times before being co-cultured with the cells. The experiment was divided into 3 groups, namely the blank control group, the normal PU textile co-cultured group and the slippery PU textile co-cultured group. The culture medium was a high sugar Dulbecco's modified Eagle's medium (High Glucose DMEM, Invitrogen, Nanjing and Biotech Development Co. Ltd) supplemented with 10% fetal bovine serum, 1% 10,000 U mL.1 penicillin and 10,000 μg mL.1 streptomycin (Gibco, Life Technolegy).The MTT assays were conducted after co-culture for 0, 1, 2, and 3 day. For the MTT assay, MTT was dissolved in PBS at 1 mg/mL and filtered through a 0.2 μm membrane. The 10% MTT solution was added into each well, and the plates were wrapped in tin foil and incubated at 37°C for another 4 h. Then the medium in each well was carefully removed, and the purple formazan products were dissolved with 400 μL of DMSO. A 100 μL solution was extracted from each well and transferred to a 96-well plate. Then the OD values were detected by a microplate reader (SYNERGY, HTX). After the data were achieved, the value of the control group at the first day was set as the standard, marked it as 100%, and then calculated the data of the other groups.

In vivo application: In vivo applications were divided into two categories, one was managing severe surface wound and the other was protecting exposed organ. A severe infected wound model was used to evaluate the effect of the textiles in wound healing. After being anesthetized, a circular skin at 2 cm in diameter on the back of the rat was removed to create a wound. Then, 100 μL of bacterial suspension was injected onto the wound area. After the

back wound has been severely infected, we used a negative pressure suction device to clean the wound. First the slippery textile of 2 cm * 2 cm was covered on the wound surface, and we covered the sponge along with a transparent film to form a closed environment. A suction cup was provided between the sponge and the transparent film to rinse and discharge the liquid. Then deionized water was constantly injected into it, and the other side kept sucking away the sewage that had been washed through the wound. In addition, the method of slippery textile used to protect exposed organs was consistent with the above steps and the abdomen was sutured after drainage. The difference was that the textile used to protect the exposed organs had a larger area to cover the entire abdominal cavity. In a typical experiment, the slippery textiles of 10 cm * 10 cm were used for viscera protection.

Characterization: Optical microscopic images of the microfibers and textiles were obtained with a stereo microscope (Olympus BX51) and recorded by a charged coupled device (CCD; SPRI F1; AOS Technologies AG). The diameter of the slippery PU microfibers was measured by software AOS Imaging Studio V3.4.2. The SEM images were obtained by using a SEM (Hitachi S-3000N). Colored photos and videos were taken by a digital camera (Canon 5D Mark II). Water contact angles were obtained at ambient temperature by using a JC2000D2 contact angle measuring system.

Author Contributions

Y.J.Z. conceived the idea; H.Z. and G.P.C. carried out the experiments, analyzed data and wrote the paper; Y.R.Y., J.H.G. and Q.T. assisted with data analysis and paper writing.

Supporting figures

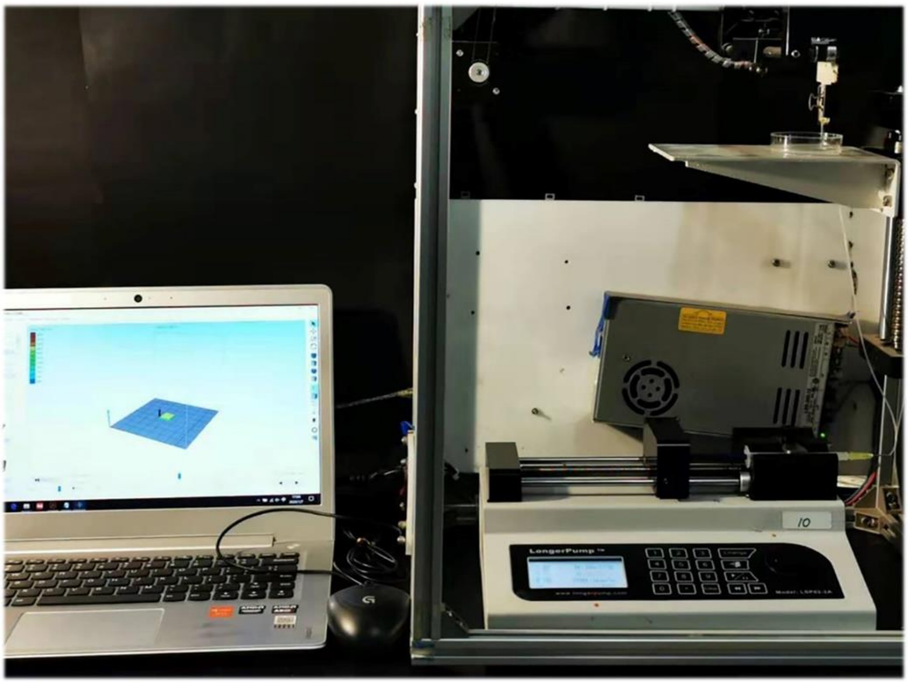

Figure S1. Digital image of the 3D-printing equipment

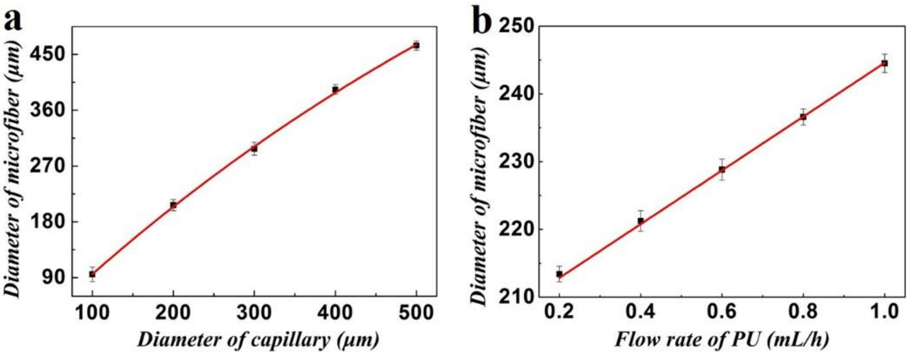

Figure S2. (a) Relationship between diameter of microfiber and that of microfluidic capillary. (b) Relationship between diameter of microfiber and flow rate of PU.

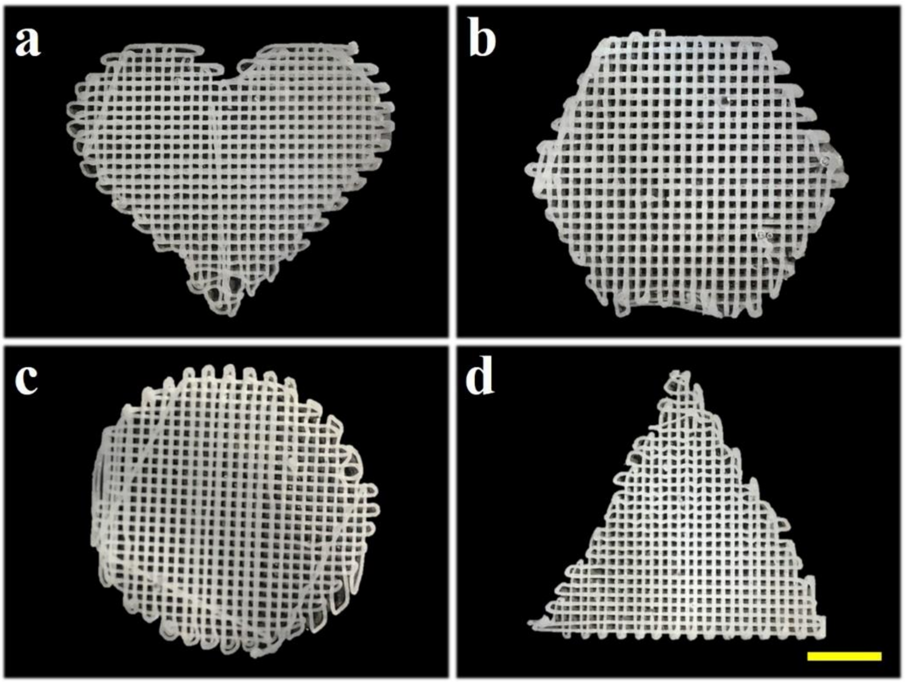

Figure S3. Digital images of different 3D PU textiles with a (a) heart, (b) hexagon, (c) circle and (d) triangle shape. Scale bar is 5mm

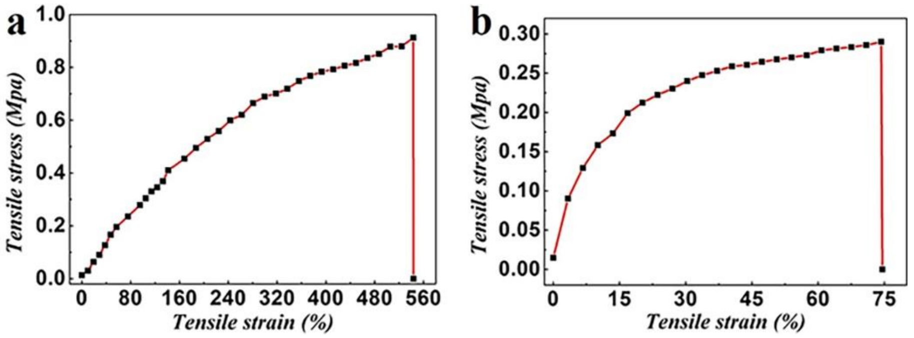

Figure S4. (a) Stress-strain test of slippery PU microfiber. (b) Stress-strain test of solid PU microfiber.

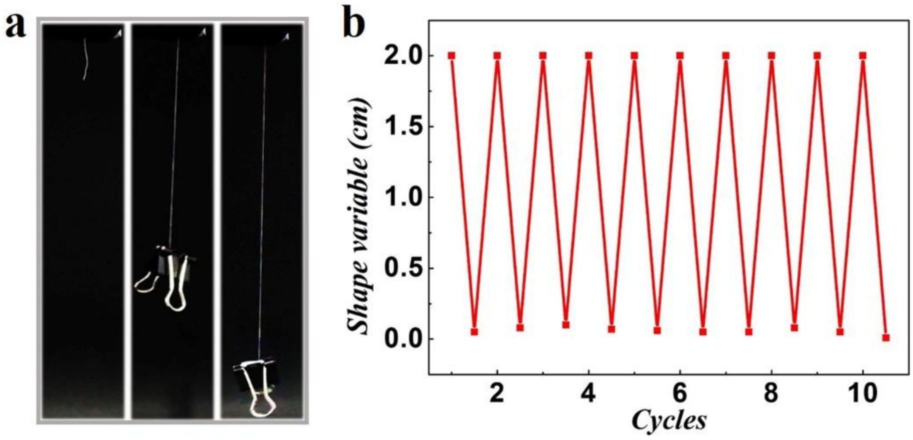

Figure S5. (a) Digital images of stretching process of slippery PU microfiber. (b) Records of slippery PU microfiber shape variation in intermittent strain cycles.

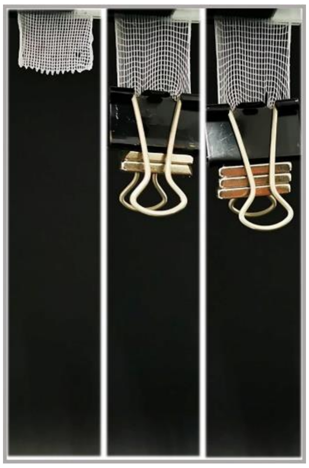

Figure S6. Digital images of the stretching process of 3D-structured PU textile.

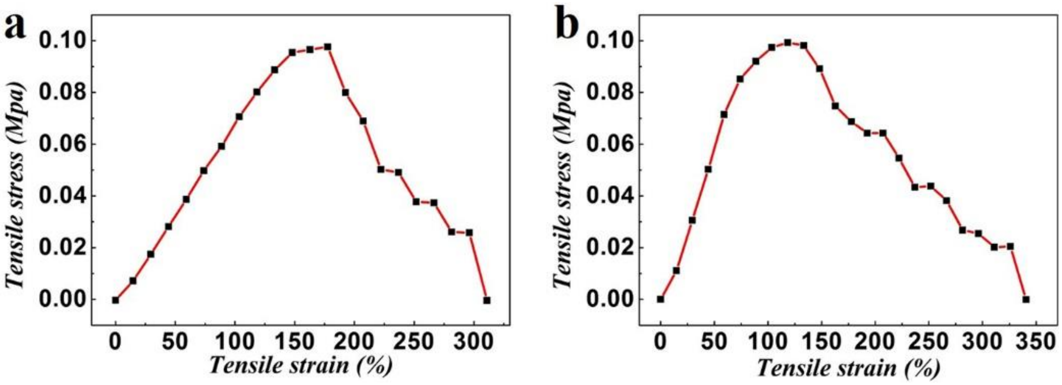

Figure S7. (a) Stress-strain test of common PU textile. (b) Stress-strain test of slippery PU textile.

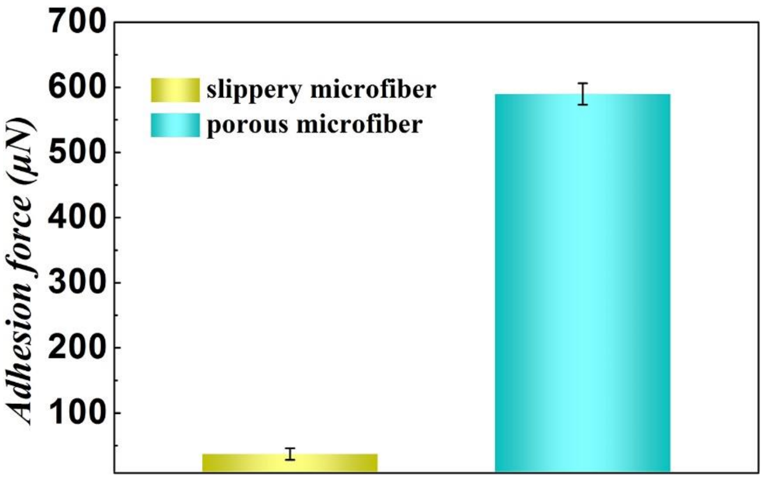

Figure S8. The adhesion force between the droplets and different microfibers.

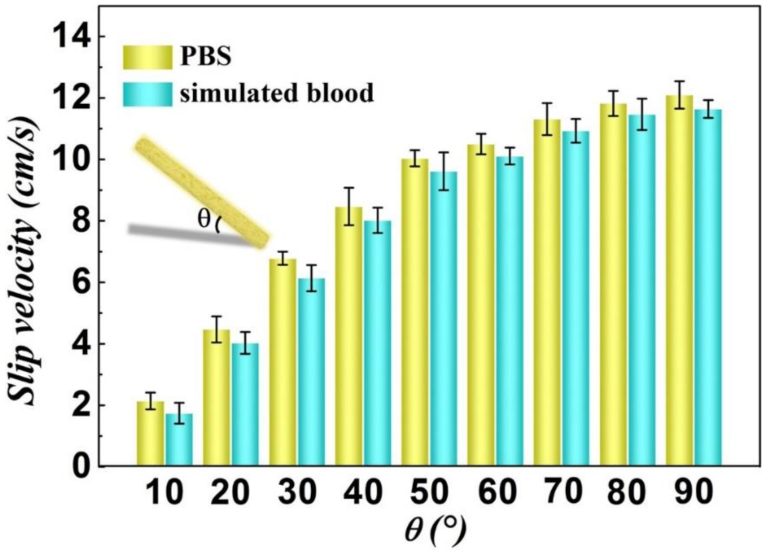

Figure S9. The relationship between slip velocity and inclination angle of different kinds of liquids.

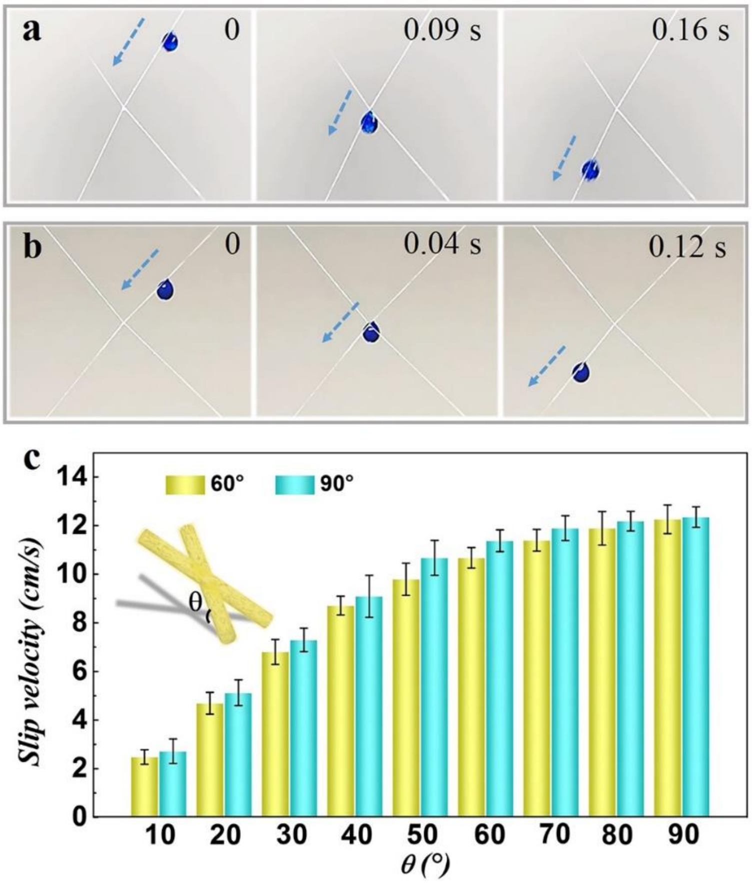

Figure S10. (a, b) Real-time images of liquid drop slip on double microfibers crossed at (a) 60°and (b) 90°. (c) The relationship between slip velocity and inclination angle of two fibers crossed at 60° (yellow) and 90° (blue).

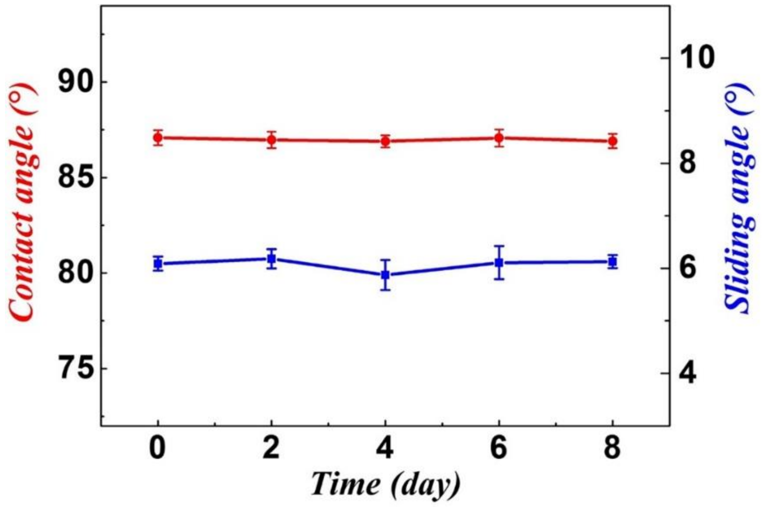

Figure S11. Characterization of contact angles and sliding angles of porous PU textile placed in PBS in a 8-day period.

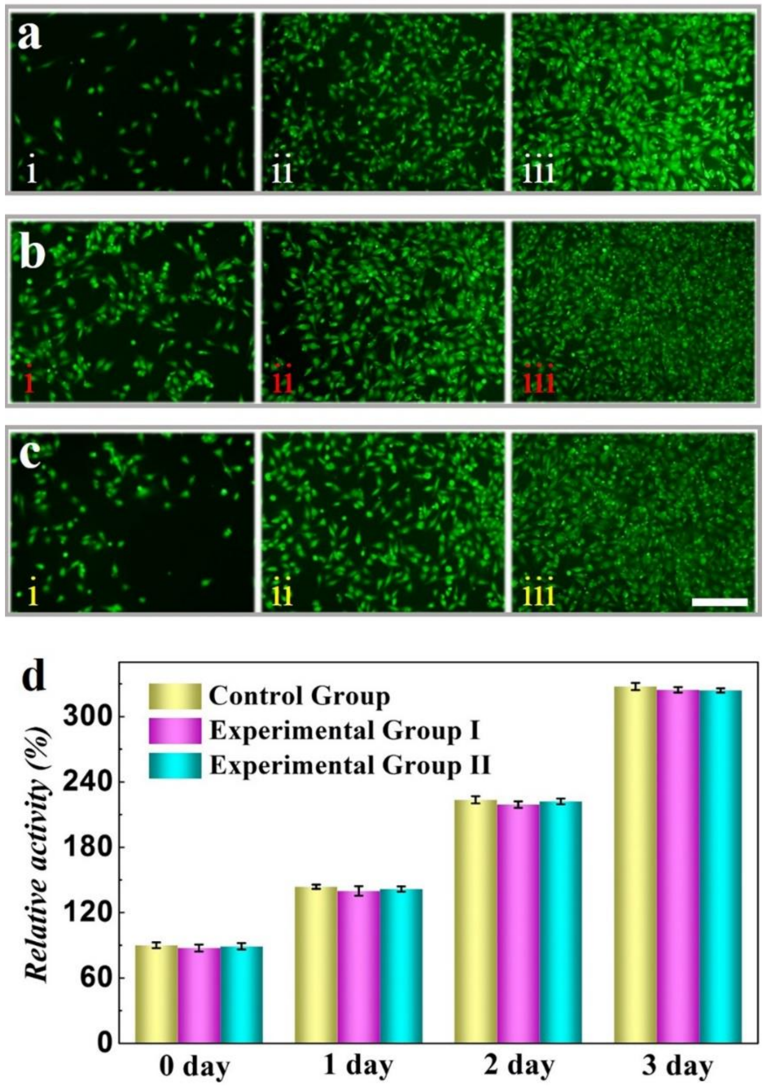

Figure S12. (a-c) Fluorescent microscopic images of 3T3 cells cultured on (a) cell culture plate, (b) common PU textile and (c) porous PU textile. (d) Statistical data of relative cell activity among control (cell culture plate), experimental group I (common PU textile) and experimental group II (porous PU textile groups). Scale bar is 200 μm.

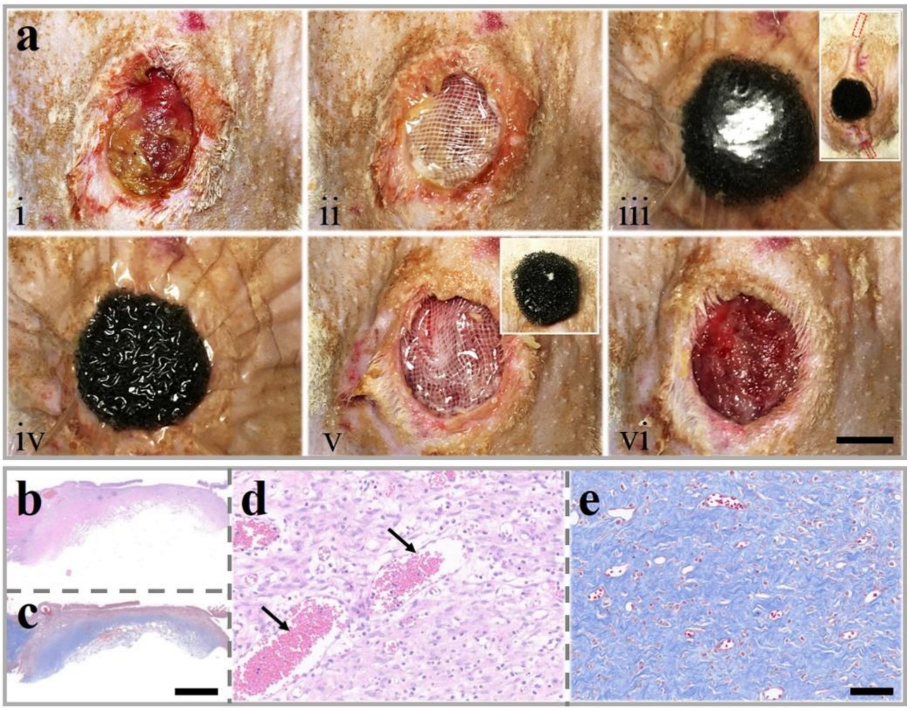

Figure S13. (a) Digital images of repair process towards dorsal wound based on slippery textile-assisted VSD. Scale bar is 1 cm. (b) H&E staining image and (d) the magnified field, (c) Masson staining image and (e) the magnified field of repaired dorsal tissue. Scale bars are 2 mm in c and 100 μm in e.

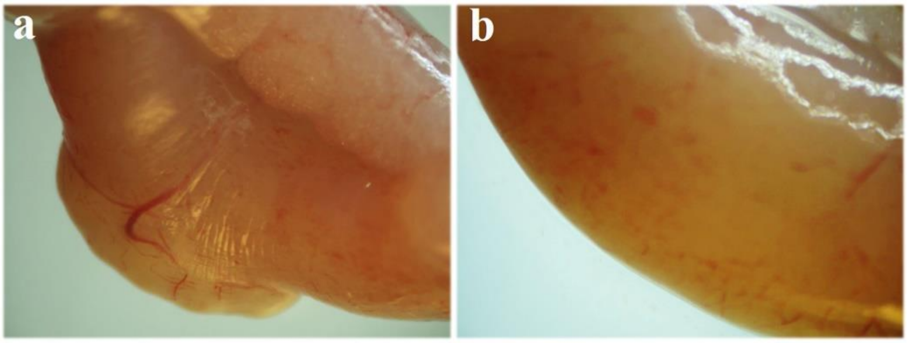

Figure S14. Optical microscopic images of (a) abraded surface and (b) protected surface of intestinal tissue.

Movie descriptions

Movie S1. The slippage of droplets on microfibers with SLIPS.
Movie S2. The wettability of the slippery textile.
Movie S3. The slippery textile for versatile wound management applications.
